# Transcriptional changes after herpes simplex virus type 1 infection in human trabecular meshwork cells

**DOI:** 10.1371/journal.pone.0217567

**Published:** 2019-05-28

**Authors:** Jin A. Choi, Hyun-hee Ju, Ju-Eun Kim, Seon-Kyu Kim, Donghyun Jee, Jiyoung Lee, Chan Kee Park, Soon-young Paik

**Affiliations:** 1 Department of Ophthalmology and Visual Science, St. Vincent's Hospital, College of Medicine, The Catholic University of Korea, Seoul, Republic of Korea; 2 Department of Microbiology, College of Medicine, The Catholic University of Korea, Seoul, Republic of Korea; 3 Personalized Genomic Medicine Research Center, Korea Research Institute of Bioscience and Biotechnology, Daejeon, Korea; 4 Department of Ophthalmology and Visual Science, Seoul St. Mary’s Hospital, College of Medicine, The Catholic University of Korea, Seoul, Republic of Korea; Cincinnati Children's Hospital Medical Center, UNITED STATES

## Abstract

**Background:**

Herpes simplex virus type 1 (HSV-1) is causative for hypertensive anterior uveitis. Trabecular meshwork (TM) cells, which are the key cells regulating intraocular pressure (IOP), is considered to be the site of inflammation. We explored the profiles of genes expressed in human TM primary cells upon HSV-1 infection.

**Methods:**

Human TM cells were infected with HSV-1 and total RNA was isolated. The global transcriptional gene network analyses were performed in mock-infected and HSV-1 infected TM cells. Using ingenuity pathway analysis, we determined the key biological networks upon HSV-1 infection. The results of microarray analyses were validated using quantitative PCR.

**Results:**

TM cells had a high susceptibility to HSV-1 infection. HSV-1 induced transcriptional suppression of many components related to fibrosis in TM cells. The top biological network related to the genes which were significantly altered upon HSV-1 infection was organismal injury and abnormalities involving TGF-β1 and PDGF-BB. The results of PCR analyses for selected molecules were found to be in good agreement with the microarray data. HSV-1-infected TM cells showed an 80-fold increase in the expression of PDGF-BB, which was further increased by treatment with TGF-β1. HSV-1 also induced a 4-fold increase in the expression of the monocyte chemoattractant protein (MCP)-1, the downstream molecules of PDGF-BB.

**Conclusions:**

In human TM cells, HSV-1 induced transcriptional suppression of many components related to fibrosis and enhanced expression of both PDGF-BB and MCP-1. Our study may provide a novel mechanism for the pathogenesis of HSV-1 infection in TM cells.

## Introduction

Herpes virus is one of the most common etiologies of acute, recurrent, and chronic anterior uveitis worldwide.[1] Herpes simplex virus (HSV) type 1 is ubiquitous and has a characteristic feature of life-long latency following primary infection. It causes various ocular problems such as conjunctivitis, keratitis, and uveitis.

Herpes anterior uveitis is usually characterized by a unilateral disease with granulomatous keratic precipitates, iris atrophy, and elevated intraocular pressure (IOP).[1, 2] It has been reported that 50%–90% of patients with herpetic uveitis develop elevation of the IOP, which was initiated at the start of uveitis, and frequent recurrences results in irreversible glaucomatous optic nerve damage.[3]

Trabecular meshwork (TM) cells are thought to be the site of inflammation in herpetic hypertensive uveitis.[4] HSV-1-appears to induce acute inflammation in TM cells, resulting in severe elevation of IOP at the time of inflammation, as shown from the thick and edematous trabecular band in an HSV-1 infection animal model.[5] However, the sudden elevation of IOP at the time of uveitis usually subsides after the cessation of inflammation. In this regard, it is probable that HSV-1 infection affects the IOP regulation mechanisms, in the early stages of the disease.

In global transcriptome analyses, HSV-1 was shown to initiate expression of IL-6 induced inflammatory mediators, whereas HSV-1 provoked antigen presentation-related inflammatory responses.[6, 7] Different host species and cells may exhibit different cellular responses to viral infection. Despite the clinical significance of HSV anterior uveitis, the pathogenesis and molecular mechanism of HSV-1 infection in TM cells is currently unclear. Elucidating the cellular responses of TM cells during HSV-1 infection may therefore provide insight into the precise mechanism responsible for the elevation of IOP induced by HSV-1.

To characterize the pathophysiological changes of TM cells upon HSV-1 infection, we performed a gene microarray analysis that simultaneously identified numerous differentially expressed genes.

## Materials and methods

### Cells and viruses

The study protocols were approved by the Institutional Review Board at the Catholic University of Korea in accordance with the Declaration of Helsinki for experiments involving human tissues and samples (local IRB No.VC18ZNSI0062). HSV-1 clinical strain NCCP no. 43002 was obtained from the National Culture Collection for Pathogens (NCCP) provided by the Korea Centers for Disease Control and Prevention (Osong, Republic of Korea; http://www.cdc.go.kr/CDC/eng/main.jsp). The percent identities of major genes between the HSV-1 NCCP no. 43002 in comparison with HSV-1 KOS strain are provided as S1 Appendix. The virus was propagated in Vero cells cultured in DMEM with 1% fetal bovine serum. When the infected cells showed cytopathic changes, the cells as well as cell culture media were collected, sonicated, and frozen at -80°C until use. Using Vero cells, standard plaque titrations were performed.[8] Primary human TM cells obtained from ScienCell Research Labs (Carlsbad, CA). In TM cell culture medium (ScienCell Research Labs), the cells were cultured to 100% confluence,[9] and cells from the fourth to sixth passage were used. For each experiment, TM cell cultures were seeded into 6-well plates and allowed to reach to confluence at 37°C in a 5% CO_2_. To infect primary TM cells with HSV-1, the TM cells were adsorbed with the virus stock for 1 h at a multiplicity of infection (MOI) of 1 or 5, and refed with fresh medium. Analyses of HSV-infected TM cells were performed 12 h after infection. In some experiments, 0.6 nM of recombinant human transforming growth factor-β1 (TGF-β1; R&D Systems, Minneapolis, MN, USA) was applied to infected and mock-infected TM cells. All cell culture experiments were performed after serum starvation.

### Viral DNA replication assays

HSV-1 viral DNA of mock-infected and infected at a MOI 1 or 5 was harvested at 12 h and 2 days after infection and isolated from Vero and TM cells. A Qiagen column (QIAmp DNA Mini Kit; Qiagen, Hilden, Germany) was used for polymerase chain reaction (PCR) analyses. We quantified the replicated viral DNA by the real-time PCR using a HSV-1 DNA polymerase primer (forward: 5'-CATCACCGACCCGGAGAGGGAC-3' and reverse: 5'-GGGCCAGGCGCTTGTTGGTGTA-3') in triplicate. The probes were also quantitated as previously described.^11^ We used β-actin as an internal control for the input DNA, and real-time PCR with β-actin primers was also performed.

### Microarray

TM cells were infected with purified HSV-1 at a MOI of 1, and mock-infected TM cells were used as controls. From the TM cells, total RNA was isolated 12 h after infection using the RNeasy Mini Kit (Qiagen). Gene expression data were generated using Affymetrix Human Gene 2.0 ST arrays (Affymetrix, Santa Clara, CA, USA). We analyzed the data with Robust Multichip Analysis using Affymetrix default analysis settings and global scaling as the normalization method. Then we analyzed the normalized and log transformed intensity values using GeneSpring GX 13.1.1 (Agilent Technologies, Santa Clara, CA, USA). Hierarchical clustering data were clustered groups that behaved similarly across experiments using the Cluster 3.0 program. The clustering algorithm involved the Euclidean distance with average linkage.

### Quantitative PCR analyses

Total RNA was extracted from TM cells from mock-infected or infected with HSV-1 at a MOI 1 of 1 and/or stimulated with recombinant active TGF-β1 at 15 ng/ml (0.6 nM) for 12 h using the RNeasy Mini Kit (Qiagen, Valencia, CA, USA). The RNA was reverse transcribed using a cDNA synthesis kit (PrimeScript RT Reagent Kit, Takara Bio, Kusatsu, Japan) according to the manufacturer’s instructions. Using a Roche Diagnostics LightCycler 2.0 Real-Time PCR System (Roche, Mannheim, Germany), the relative expression levels of mRNA were determined as previously described.[10] Reactions for each sample were run in triplicate, cycle thresholds were normalized to β-actin expression, and comparative quantitation was performed (LightCycler software, version 4.1, Roche). Only individual PCR samples with single peak dissociation curves were selected for data analysis. Table 1 shows the sequences of the primer sets. To ensure equal loading and amplification, all products were normalized to a β-actin as an internal control. The results are the averages of three independent experiments.

**Table 1:** Sequence for forward and reverse primer sets used for real-time PCR.

Amplification	Forward primer	Reverse primer
α-SMA	5′ -GACAATGGCTCTGGGCTCTGTAA-3′	5′ -CTGTGCTTCGTCACCCACGTA-3′
collagen1A	5′ -GGAATGAAGGGACACAGAGG-3′	5′ -TAGCACCATCATTTCCACGA-3′
CTGF	5′ -CTCCTGCAGGCTAGAGAAGC-3′	5′ -GATGCACTTTTTGCCCTTCTT-3′
fibronectin	5′ -CTGGCCGAAAATACATTGTAA-3′	5′ -CCACAGTCGGGTCAGGAG-3′
TGF-β1	5’ -GAGCCTGAGGCCGACTACTA-3’	5’-GGGTTCAGGTACCGCTTCTC-3’
TGF-β2	5’ -GTCGCGCTCAGCCTGTCT-3’	5’-CCTCGATCCTCTTGCGCAT-3’
PDGF-B	5’ -TGATCTCCAACGCCTGCT-3’	5’-TCATGTTCAGGTCCAACTCG-3’
MCP-1	5’ -CTGAAGCTCGTACTCTC-3’	5’-CTTGGGTTGTGGAGTGAG-3’
β-actin	5′ -GTCCACCTTCCAGCAGATGT-3′	5′ -AAAGCCATGCCAATCTCATC-3′

α -SMA: smooth muscle actin; CTGF: connective tissue growth factor; TGF: transforming growth factor; PDGF: platelet derived growth factor; MCP: Monocyte chemoattractant protein.

### Statistical analysis

Significant differences between groups were evaluated by using the Student’s *t-*test. One-way analysis of variance was used for comparison of results between three groups. *P* < 0.05 was regarded as a significant difference between two groups.

## Results

### Synthesis of viral gene transcripts in human TM and Vero cells

**Fig 1:**
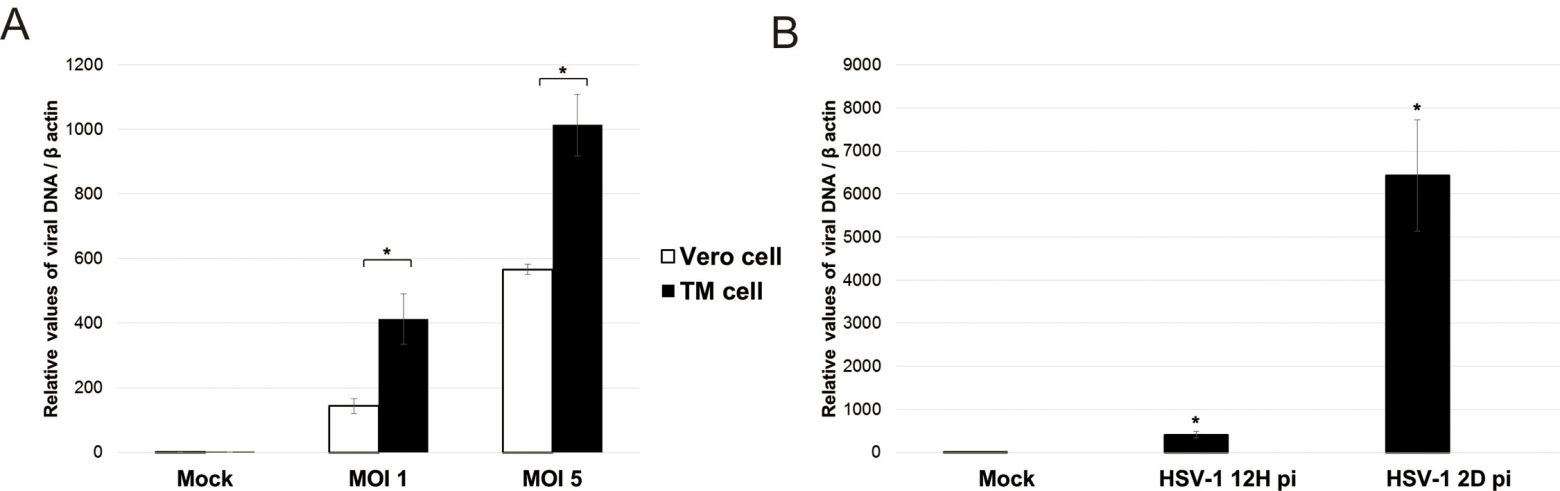
Accumulation of viral transcripts after human HSV-1 infection in human trabecular meshwork cells (TM) and Vero cells. (A) Comparisons of accumulation of viral transcripts in human TM cells and human Vero cells at 12 hours pot infection. HSV-1 was infected at a multiplicity of infection (MOI) 1 or 5 in human TM cells and Vero cells. (B) Serial changes of viral transcripts at 12 hours and 2 days post infection in human TM cells. Viral transcripts were processed for qPCR analysis of viral DNA accumulation using HSV-1 DNA polymerase primer. Real-time PCR with β -actin primers was performed to serve as an internal control for input DNA. Data are the averages of three independent DNA samples from the infected cells. Values are mean ± standard error.

To investigate whether human TM cells supported HSV-1 expression, we compared viral gene transcripts in human TM cells with those in Vero cells after HSV-1 infection with an MOI of 1 or 5 at 12 h after infection. There was a 2-fold increased expression level of HSV-1 DNA polymerase mRNA in the HSV-1 infected TM cells compared with the Vero cells (*P* < 0.05 at MOI 1 and 5, respectively; Fig 1A). The expression of HSV-1 DNA polymerase mRNA was exponentially increased at 2 days after infection in TM cells (Fig 1B).

The Ct values and RT-PCR results of β-actin in Vero cells and TM cells shows that the expression levels of house-keeping gene is not altered by HSV-1 at 12 h and 2 days post infection. The cell viability assay shows that the proportion of the live TM cells was 80% with an MOI of 1 at 12 h after infection (S3 Appendix).

### Transcriptome analysis of the HSV-1-infected TM cells

**Fig 2:**
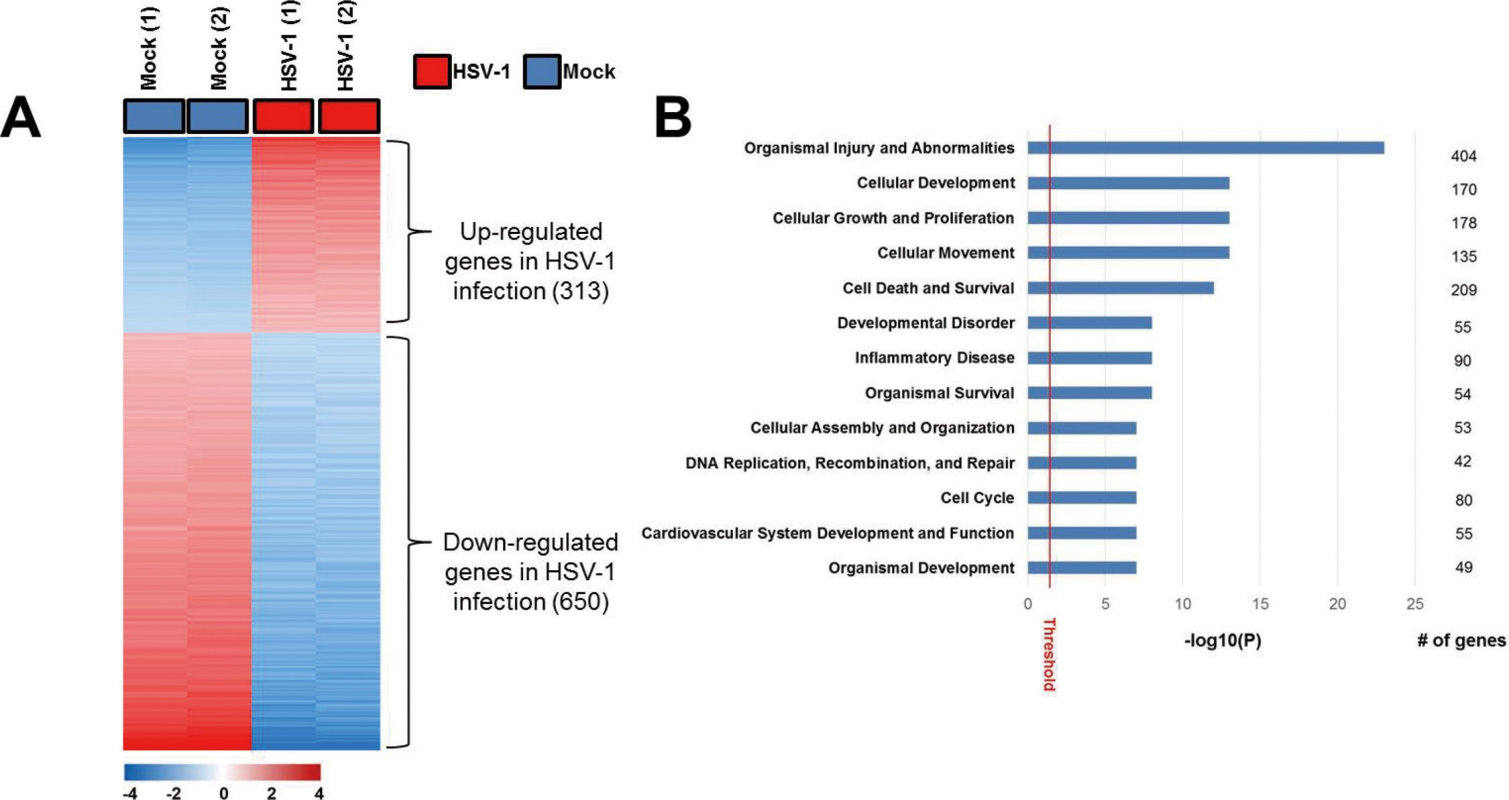
Clustering analysis of HSV-1 infection induced transcriptome in human TM cells and most significant biologic processes underlying the differentially expressed genes upon HSV-1 infection. **(**A) Gene expression pattern of the samples involved in MOCK and HSV-1 groups (n = 4) A total of 3,280 genes having more varied expression change across the samples were selected for a cluster analysis [standard deviation (SD) > 0.7]. The expression levels are color-coded (red, activated; blue, suppressed) (B) IPA was used to investigate biologic processes significantly associated with differentially expressed genes.

To determine the molecular characteristics compatible with HSV-1 infection, a total of 3,280 genes having more varied expression changes across samples were selected for a cluster analysis [standard deviation (SD) > 0.7; Fig 2A], and a total of 963 statistically significant genes were selected by two sample *t*-tests (*P* < 0.01, 2-fold or more difference in expression). In the analysis, 313 genes were found to be upregulated and 650 genes were downregulated in HSV-infected TM cells relative to mock-infected TM cells. A gene set enrichment test was carried out by the Ingenuity Pathway Analysis (IPA) tool. When applying the genes to IPA, genes involved in organismic injury and abnormalities, cellular development, cellular growth and proliferation, cellular movement, and cell death and survival were strongly enriched (Fig 2B).

Total of 46 upstream regulators was identified to be significant (P < 1 x 10^−3^) in the IPA upstream regulator analysis. Among these, the most significant upstream regulator was TGF-β1, and one of the activated regulators was the PDGF-BB (Table 2).

**Table 2:** Prediction of significant upstream regulators in the HSV-1 infected TM cells.

Upstream Regulator	Expr Log Ratio	Molecule Type	Activation z-score	p-value of overlap
TGFβ-1	-1.378	growth factor	-0.856	2.27E-11
estrogen receptor		group	2.976	5.44E-10
TP63		transcription regulator	-0.545	0.000000164
TP53		transcription regulator	1.265	0.000000215
SYVN1		transporter	-2.286	0.000000249
FOXO1		transcription regulator	-0.793	0.000000913
HIF1A		transcription regulator	-3.361	0.00000128
ESR1		ligand-dependent nuclear receptor	-1.712	0.00000202
SPDEF		transcription regulator	3.317	0.00000265
ITGAV	-2.168	transmembrane receptor	-1.741	0.00000377
FBN1		other	2.425	0.00000487
CDKN1A		kinase	1.174	0.00000767
CHI3L1		enzyme		0.0000134
NR3C1		ligand-dependent nuclear receptor	-0.787	0.0000191
FOXO3		transcription regulator	0.927	0.0000241
YAP1		transcription regulator	0.461	0.0000345
MYC		transcription regulator	-0.894	0.0000347
TNF		cytokine	-0.073	0.0000418
ERBB2		kinase	-3.477	0.0000481
STK11		kinase	0.233	0.0000541
SMARCA4		transcription regulator	-2.634	0.0000572
Jnk		group	-1.612	0.0000642
ATM		kinase	-0.492	0.0000646
BRCA1		transcription regulator	-0.512	0.0000649
ERK		group	-0.366	0.0000837
IL1A		cytokine	-1.05	0.0000875
CD44		other	-1.676	0.000102
NUPR1		transcription regulator	3.795	0.000107
ZNF652		other		0.000111
PDGF BB		complex	2.887	0.000114
S100A6		transporter	-1.89	0.000118
CRNDE		other	0.707	0.000125
KDM5B	1.63	transcription regulator	3.191	0.00013
CD24		other	-1.134	0.00013
MITF		transcription regulator	-2.496	0.000178
HEXIM1	1.903	transcription regulator	2	0.000215
Lh		complex	-0.573	0.000227
TAF4B		transcription regulator		0.000316
E2F4		transcription regulator		0.00036
EPAS1		transcription regulator	-0.588	0.000369
HDL		complex	-1.992	0.000374
TGFBR1		kinase	1.513	0.000611
KLF6		transcription regulator	0	0.000719
SMAD2		transcription regulator	0.085	0.000891
SMAD7		transcription regulator		0.000913
CIP2A		other	-0.333	0.000941

**Fig 3:**
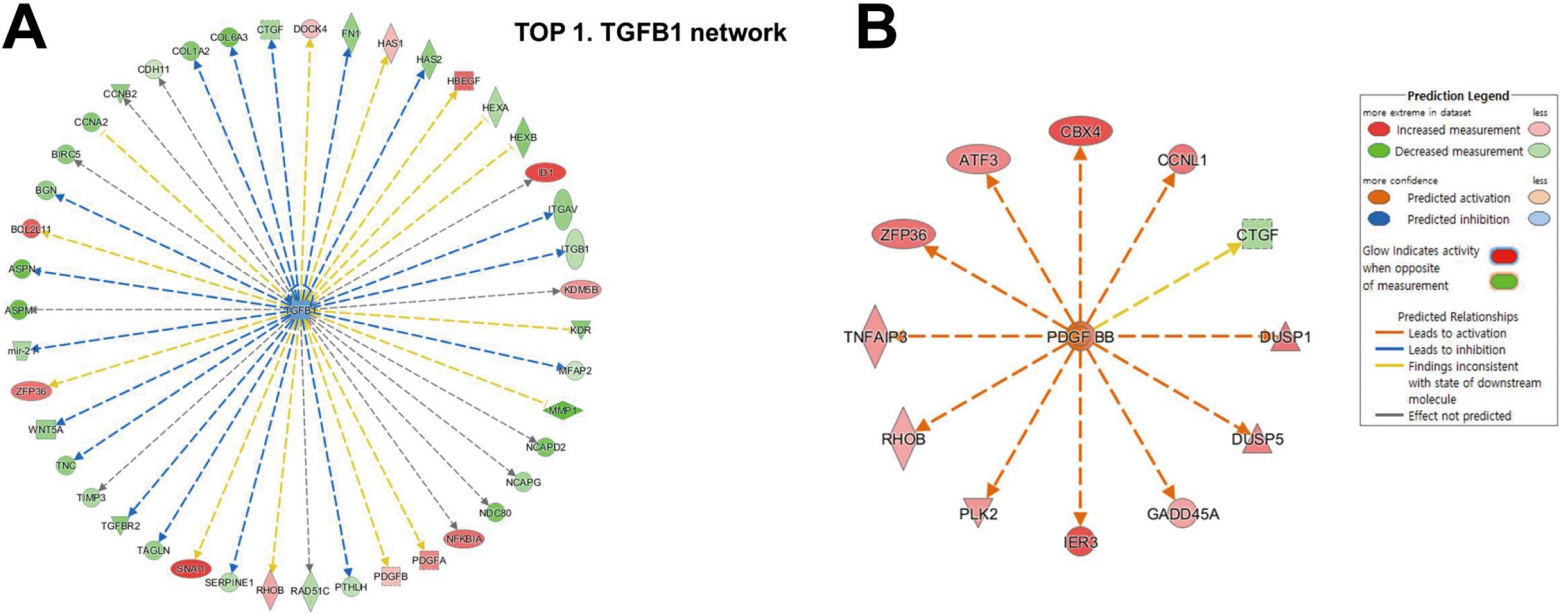
Biologic pathway identified from RNA sequencing data in HSV-1 infected TM cells. Predicted involvement of TGF-β1 signaling pathway (A) and predicted activation of PDGF-BB signaling pathway (B) by molecule activity predictor analysis of IPA in HSV- infected versus mock infected TM cells. Gene networks enriched with genes associated with HSV-1 infection in human TM cells. Red and green color represents the up- and down-regulated genes in the HSV-1 infected TM cells, respectively. The intensity of color indicates the degree of over- or under-expression. Functional and physical interactions between the genes and the direction of regulation reported in the published literature are represented by each line and arrow.

Fig 3 shows the predicted involvement of TGF-β1 signaling pathway (Fig 3A) and activation of PDGF-BB signaling pathway (Fig 3B) by molecule activity predictor analysis of IPA in HSV- infected versus mock infected TM cells.

### Expression of molecules induced by HSV-1 infection and treatment with TGF-β1

**Fig 4:**
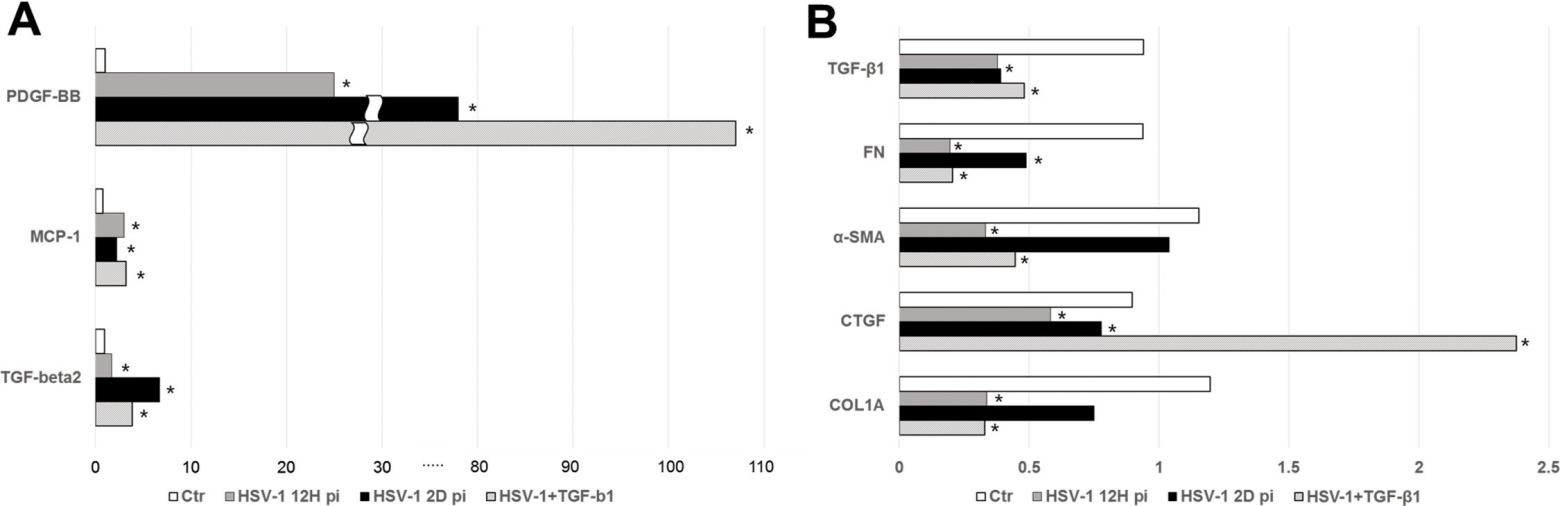
Real-time quantitative PCR according to HSV-1 infection and/or treatment with TGF-β1 at 12 hours and 2 days post infection. Cells were infected with HSV-1 at a multiplicity of infection (MOI) 1. (A) Up-regulated molecules induced by HSV-1 infection (B) Down-regulated molecules induced by HSV-1 infection. Expression of PDGF-B, MCP-1, and TGF-β2 were significantly up-regulated and the expression of TGF-β1, fibronectin, α-smooth muscle actin, collagen-1A, and connective tissue growth factor were significantly down regulated by HSV-1 infection.(*P < 0.05 vs. transcripts from the unstimulated mock infection).

To validate the gene expression profile identified by the microarray, we compared the expression of TGF-β1, PDGF-B, and MCP-1, the downstream molecules of PDGF-B, and other fibrogenic molecules. HSV-1-infected TM cells showed a 20-fold and a 80-fold increases in the expression of PDGF-B (*P* < 0.001) at 12 hours and 2 days after infection, which was further increased by treatment with TGF-β1 (*P* = 0.035; Fig 4A). HSV-1 infection also induced a 4-fold increase in the expression of MCP-1 at 12 hours post infection (*P* <0.001; Fig 4A). However, HSV-1 infection caused generalized inhibition of fibrogenic molecules such as TGF-β1, α-smooth muscle actin (SMA), fibronectin, connective tissue growth factor (CTGF) and collagen (COL)-1A at 12 hours and 2 days after infection (Fig 4B). However, TGF-β2 was a 1.7-fold and a 6.7-fold increased by HSV-1 infection at 12 hours and 2 days after infection, respectively (*P* < 0.001, both, Fig 4A).

## Discussions

Many growth factors and cytokines are found in the aqueous humor (AH). Among them, sufficient levels of TGF-β exist in the AH for physiologic function of eyes.[11] However, TGF-β generally exerts negative regulatory effect on innate and adaptive immune response. TGF-β signaling is known to result in increased HSV-1 latency.[12] Therefore, human TM cells, nourished by the aqueous humor, should provide an enriched environment for viral replication. Tiwari et al. reported that primary human trabecular meshwork cells were permissive for HSV-1 infection, and TM cell expresses high level of anti-herpesvirus entry mediator, which facilitates viral infection.[13] In accordance with their study, we also found a 2-fold increased expression level of viral gene transcripts in the HSV-1 infected TM cells compared with the Vero cells (Fig 1A), which were exponentially increased at 2 days after infection (Fig 1B).

Using gene set enrichment, we found that genes related with organismal injury and abnormalities were commonly expressed (Fig 2B). It is known that HSV-1 induces significant decrease in the transcription of host genes, caused by silencing infected cells and preventing them from responding to the virus infection. [14–16] In this study, we also showed that 67.5% of the transcripts were downregulated (Fig 3 and Table 2), which seems to be mediated by silencing of the host genes by viral proteins. The qPCR also showed the transcriptional downregulation of fibrogenic genes for TGF-β-1, fibronectin, α-SMA, collagen 1A, and CTGF (Fig 4B). Contrary to our results, Terasaka et al.[7] reported that HSV-1 infection showed global transcriptional activation in corneal epithelial cells, and in the HEp-2 epithelial cells.[17] Human TM cells, the key cells regulating IOP, are metabolically active with large amounts of endoplasmic reticulum, ribosomes, and mitochondria.[18] The significant downregulation of host genes in TM cells may therefore result in IOP dysregulation.

Using transcriptome analyses, the TGF-β1 network was found to be the most probable candidate for upstream regulation associated with HSV-1 infection (Table 2). The IPA showed that the top biological network related to the genes that were significantly modified upon HSV-1 infection was organismal injury and abnormalities involving TGF-β1 and PDGF-BB. (Figs 2A and 3). The predicted activation of PDGF-BB signaling pathway in network analyses was validated in the qualitative PCR results, in which PDGF-B mRNA expression showed a 20-fold increase upon HSV-1 infection (Fig 4A). It has been established that HSV evokes enhanced production of VEGF-A, resulting in corneal neovascularization.[19] Along with VEGF, PDGF, distributed in fibroblast, vascular smooth muscle cells, endothelial cells, RPE cells, and macrophage, has an important role in the angiogenesis.[20] It is known that angiogenesis is tightly controlled by endothelial-stromal interactions involving PDGF-B, VEGF, and TGF-β.[20] Ohira et al., who investigated factors associated with aqueous pro-inflammatory growth factors and cytokines in eyes with uveitic glaucoma, reported that PDGF-BB was significantly elevated in eyes accompanied by anterior chamber cells and those with infectious uveitis.[21]

Importantly, the qualitative PCR revealed that MCP-1, and the downstream molecules of PDGF-BB, were significantly elevated during HSV-1 infection (Figs 3 and 4A) Consistent with the results of our study, previous studies reported that MCP-1 was upregulated during HSV-1 ocular infection.[22] MCP-1, is one of the key mediators in inflammatory processes.[23] The aqueous level of MCP-1 was found to be significantly elevated in eyes with uveitic glaucoma, compared with those with primary open angle glaucoma (POAG).[21] MCP-1 is involved in cytoskeletal remodeling in vascular smooth muscle cell with regard to mediating cell migration, suggesting amplified contraction in human TM cells.[24] Also, MCP-1 may induce monocyte recruitment into human TM cells following HSV-1 infection, which may result in blocking of the outflow facility and elevation of the IOP. Further mechanism of PDGF-BB and MCP-1 in IOP elevation needs to be clarified.

In this study, the expression of TGF-β1 was decreased (Fig 4B), whereas TGF-β2 mRNA expression was increased with HSV-1 infection with the progression of time (Fig 4A). This differential expression of TGF-β1 and TGF-β2 was also reported by Nie et al.,[25] in which HSV-1-infected corneal epithelial cells expressed significantly decreased TGF-β1, but showed no noticeable changes in the levels of TGF-β2 and TGF-β3. It is known that the patients with POAG accompany significantly elevated levels of TGF-β2 in the aqueous humor.

Contrary to the HSV-1 infection, *in vivo* infection of cytomegalovirus (CMV) enhances the secretion of TGF- β1 in fibroblasts, osteosarcoma cells and astrocytes.[26, 27] TGF-β1 and epithelial-mesenchymal transformation-associated genes were shown to be upregulated following CMV infection by activating TGF-β1.[28] In the previous study, we reported that CMV enhances the production of TGF-β1 in human TM cells.[10] Considering that HSV-1 induced transcriptional suppression of TGF-β1 and many components related to fibrosis in TM cells, the mechanism responsible for IOP elevation may differ according to the causative viruses.

In our study, the proportion of the live cells was nearly 80% 12 hours after infection, which are adequate for the analyses of the host gene changes (S3 Appendix). However, in the laboratory *in vitro* setting, which does not involve the defense from the host immunologic reaction, the virus is allowed to replicate exponentially and finally causes the death of the cells by the causative virus. Therefore, it is not feasible to evaluate the overall host-related pathophysiologic process of viral anterior uveitis with the in vitro HSV-1 infection model. The transient nature of clinical manifestation at the early stage of the viral anterior uveitis may involve the changes of the cytokines or infiltration / blockage by inflammatory cell or debris at the outflow facility. However, with repeated attack or progression of the disease, it would involve the loss of TM cells, which would finally lead to persistent IOP elevation, as shown in the pathogenesis of POAG.

In summary, the present study confirmed that human TM cells were permissive to HSV-1. In the TM cells, HSV-1 induced transcriptional suppression of many components related to fibrosis and enhanced expression of both PDGF-BB and the chemokine MCP-1. Understanding of the molecular characteristics compatible with HSV-1 infection in TM cells would support to provide strategy against the sudden elevation of IOP in HSV anterior uveitis.

## Supporting information

S1 Appendix The percent identities of major genes of HSV-1 between the clinical strain HSV-1 NCCP no. 43002 in comparison with HSV-1 KOS strain. (XLSX)

S2 Appendix Ct values and RT-PCR results of the house keeping gene (β-actin) in Vero cells and TM cells according the presence of HSV-1. (TIF)

S3 Appendix The cell viability assay and the morphology of the TM cells on bright-field microscopy. (TIF)
