# Supplementary figures and images for: Transcriptional changes after herpes simplex virus type 1 infection in human trabecular meshwork cells
Source: PLoS One. 2019 May 28;14(5):e0217567. doi: 10.1371/journal.pone.0217567 (PMC6538180; doi:10.1371/journal.pone.0217567)

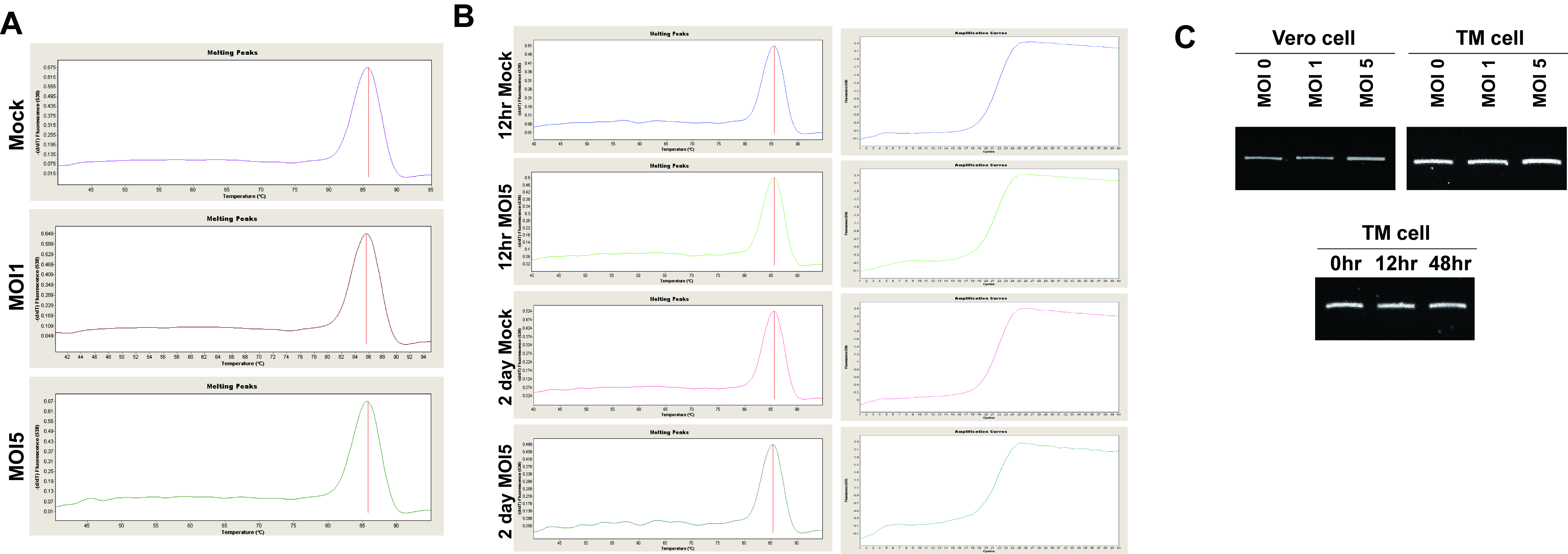

Supplement: S2 Appendix — (TIF) [file pone.0217567.s002.tif]

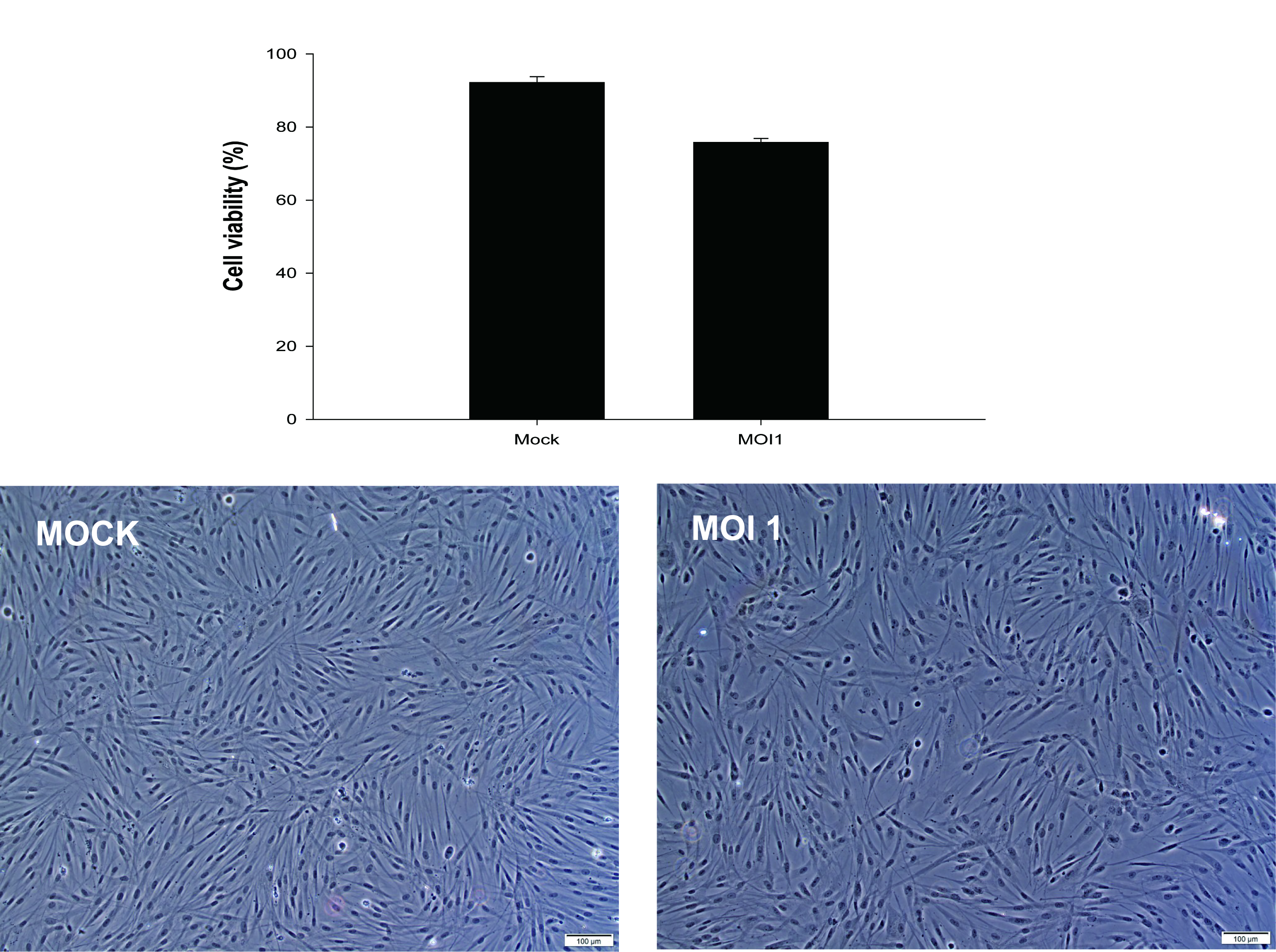

Supplement: S3 Appendix — (TIF) [file pone.0217567.s003.tif]
